# A new global anthropogenic heat estimation based on high-resolution nighttime light data

**DOI:** 10.1038/sdata.2017.116

**Published:** 2017-08-22

**Authors:** Wangming Yang, Yibo Luan, Xiaolei Liu, Xiaoyong Yu, Lijuan Miao, Xuefeng Cui

**Affiliations:** 1 Master of science, State Key Laboratory of Earth Surface Processes and Resource Ecology, School of System Science, Beijing Normal University, Beijing, China; 2 Assistant Research Fellow, Institute of African Studies, Nanjing University, Nanjing 210000, China; 3 Lecturer, Doctor, Polar Climate System and Global Change Laboratory, Nanjing University of Information Science and Technology, Nanjing 210044, China; 4 Lecturer, Doctor, College of Geography and Remote Sensing, Nanjing University of Information Science and Technology, Nanjing 210044, China; 5 Leibniz Institute of Agricultural Development in Transition Economies (IAMO), Halle (Saale), Germany; 6 Professor, Doctor, Meteorology and Climate Centre, School of Mathematics and Statistics, University College Dublin, Belfield Dublin 4, Ireland; 7 State Key Laboratory of Earth Surface Processes and Resource Ecology, School of System Science, Beijing Normal University, Beijing, China

**Keywords:** Environmental chemistry, Climate change, Energy and behaviour, Geography

## Abstract

Consumption of fossil fuel resources leads to global warming and climate change. Apart from the negative impact of greenhouse gases on the climate, the increasing emission of anthropogenic heat from energy consumption also brings significant impacts on urban ecosystems and the surface energy balance. The objective of this work is to develop a new method of estimating the global anthropogenic heat budget and validate it on the global scale with a high precision and resolution dataset. A statistical algorithm was applied to estimate the annual mean anthropogenic heat (AH-DMSP) from 1992 to 2010 at 1×1 km^2^ spatial resolution for the entire planet. AH-DMSP was validated for both provincial and city scales, and results indicate that our dataset performs well at both scales. Compared with other global anthropogenic heat datasets, the AH-DMSP has a higher precision and finer spatial distribution. Although there are some limitations, the AH-DMSP could provide reliable, multi-scale anthropogenic heat information, which could be used for further research on regional or global climate change and urban ecosystems.

## Background & Summary

Anthropogenic heat is wasted heat in the form of sensible and latent heat (http://www.ametsoc.org/amsedu/) that is released to the urban canopy mainly by means of heating and cooling, running appliances, transportation, and industrial processes, which convert energy into anthropogenic heat^1–4^. Nearly 70% of energy is consumed within cities occupying a mere 2% of the Earth’s surface area, and future scenarios indicate that global primary energy consumption will rise 1.6 times (864.7 quadrillion kJ) from 2010 to 2040 (http://www.worldenergyoutlook.org/). Although anthropogenic heat accounts for only 1% of the greenhouse gas forcing, it causes the majority of regional warming, such as urban heat islands^1,2^, urban boundary heights, and hourly intensity of precipitation at the city level^2–5^, especially at night. Anthropogenic heat reduces the intensity of precursor species (NOx) in the urban atmosphere due to the dilution, increases concentrations of near surface ozone^6,7^, and raises risks for morbidity and mortality of the residents through increasing the air temperatures in urban areas^8^. Meanwhile, many studies indicate that anthropogenic heat increases the temperature, especially in urban regions and cold seasons, which affects the Asian winter monsoon circulation^9^ and disrupts the normal global atmospheric circulation patterns^10^. Considering the remarkable growth of energy use expected in the future^11^, anthropogenic heat will play an increasingly important role in the radiative forcing of the atmosphere, global climate changes, and urban ecosystem impacts.

Therefore, calculating a high spatial resolution global scale anthropogenic heat flux (AHF) dataset is necessary for studying climate impacts. According to the characteristics of anthropogenic heat, three main approaches have been developed: observations, detailed statistical models (building/traffic energy modelling), and inventory approaches. As a classical method, the inventory approach is used globally to estimate global-scale anthropogenic heat^12,13^, using the assumption that any heat emitted by energy consumption is completely converted to anthropogenic heat. The observation approach includes an energy budget residual approach^14–16^ and in situ eddy covariance observations^13^. Statistical detailed models, such as the town energy budget (TEB) or urban canopy model^17^, have been developed according to building materials, heights, equipment, as well as transportation equipment^18–20^. Each approach has its own advantage; however, the inventory approach is more efficient for large-scale studies than the other two, which are limited by their demand categories and modelling resolutions. Some attempts have been made at developing a global AHF dataset. In the first global anthropogenic heat dataset (released in 2009, referred to as the Flanner study hereafter), Flanner proportionally downscales the total country-level non-renewable energy consumption to a 2.5-minute resolution according to the spatial population density^21^. However, this global dataset has unreliable information for regions where the population density does not have a clear statistical relationship with economic development and energy consumption, such as in China^22^. To overcome the limitations of Flanner’s study, in Chen’s study, a simple model was built based on the relationship of nighttime light data from the US Air Force Defense Meteorological Satellite Program/Operational LineScan System (DMSP/OLS) and energy consumption^23^. However, information in downtown areas has lower reliability due to the saturation and temporal fluctuation characteristics of nighttime light data^24^. In this study, we attempted to develop an updated method of generating an improved precision and high spatial resolution global anthropogenic heat dataset. Normalized difference vegetation index (NDVI) was used to calibrate DMSP/OLS data, and then used as a proxy to downscale the country-level inventory data to a 1×1 km spatial resolution. Using statistical methods, we validated the feasibility of the method and the reliability of the AH-DMSP dataset.

AH-DMSP shows that the majority of anthropogenic heat is mainly produced in the developed regions-the eastern part of North America, Western Europe, the eastern and southern parts of Asia as a result of the high intensity and magnitude of human activities in these regions. All of the annual maximum values appear in urban regions. Changes in anthropogenic heat between 1990 and 2010 were significant in the metropolis regions of both the developing and developed countries.

## Methods

### Data

#### DMSP/OLS nighttime light data

Cloud-free composite DMSP/OLS data from 1992 to 2010 were acquired from the National Oceanic and Atmospheric Administration’s (NOAA) National Geophysical Data Center^25^. Three of four satellites from the DMSP carry the OLS in low-altitude polar orbits to record nighttime data. The DMSP/OLS has the unique capability to detect low levels of visible near infrared (VNIR) radiance at night. With the OLS ‘VIS’ band data, it is possible to detect clouds illuminated by moonlight, plus lights from cities, towns, industrial sites, gas flares, and ephemeral events such as fires and lightning-illuminated clouds. Data values range from 1–63. Areas with no cloud-free observations are represented by the value 255. The stable average nighttime lights detected by DMSP/OLS contain the lights from cities, towns, and other sites with persistent lighting, including gas flares. Ephemeral events, such as fires, were discarded^26,27^. Background noise was identified and replaced with zero values. In this work, the DMSP/OLS data is regarded as a proxy to the energy consumption intensity in cities and towns, as well as in other persistent lighting sites, including gas flares (Data Citation 1).

#### Normalized difference vegetation index (NDVI) Data

Gap-filled, snow-free, Nadir Bidirectional Reflectance Distribution Function (BRFD) Adjusted Reflectance (NBAR) data derived from the Moderate Resolution Imaging Spectro radiometer (MODIS) MCD43D products were used to generate NBAR-NDVI time series. For each year 2001–2010, the annual mean NDVI was calculated for each pixel^27,28^. To calibrate DMSP/OLS data, we prefer the annual mean NDVI to the annual maximum NDVI, because the former is more stable and less sensitive to seasonal and intra-annual fluctuations. To keep temporal consistency with the DMSP/OLS data, we extended the annual mean NDVI from year 2001 as constant values back to 1992, to complement the period 1992–2001. This decision is supported by the observation that there is not significant annual variation in the annual mean NDVI for urban regions during 2001–2010 (Data Citation 2).

#### Gridded population data

We obtained gridded population density data for the year 2000 which are 2.5 arc-minute grid cells of the Gridded Population of the World v3 (GPWv3), provided by the Centre for International Earth Science Information Network (Data Citation 3). The population density grids were derived by dividing population count grids by a land area grid, and represent persons per square kilometre^29,30^.

#### Statistical energy consumption data

Statistics of national primary energy consumption for 224 countries were obtained from the U.S. Energy Information Administration (EIA)^11^. The statistics include energy from the four main primary energy sources: coal, petroleum, natural gas, and renewable energy. Chinese urban level energy consumption was obtained from the Urban Statistical Yearbook for China (http://data.stats.gov.cn/easyquery.htm? cn=E0103). Gross national income (GNI) per capita for each of 224 countries was obtained from the World Bank (http://data.worldbank.org/income-level/OEC). A portion of the primary energy sources are used to produce secondary energy (for example, the coal used by a power station to generate electricity) in non-urban areas that is then consumed by urban residents at the end of the energy flow. In this case, the wasting heat in the processing procedure was not taken into consideration since it has little influence on total heat emitted. We therefore use only the four primary energy source classifications in this study.

### Methodology

**Figure 1:**
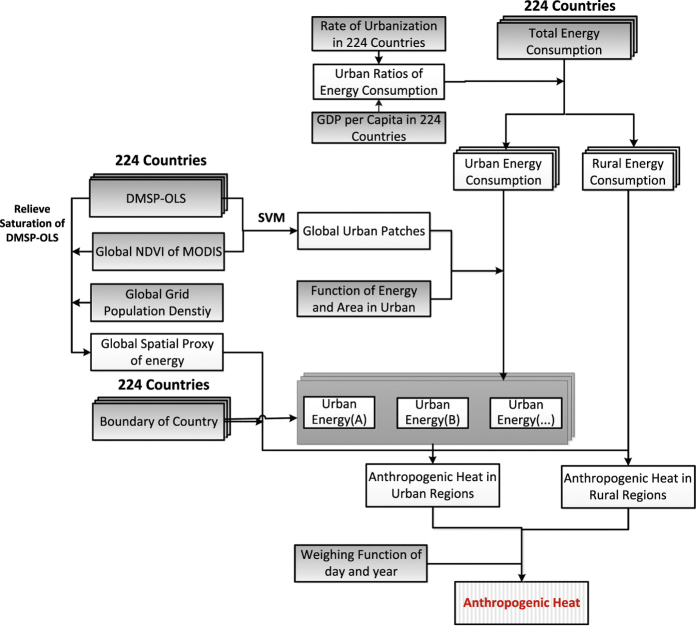
Flow chart of calculations.

**Figure 2:**
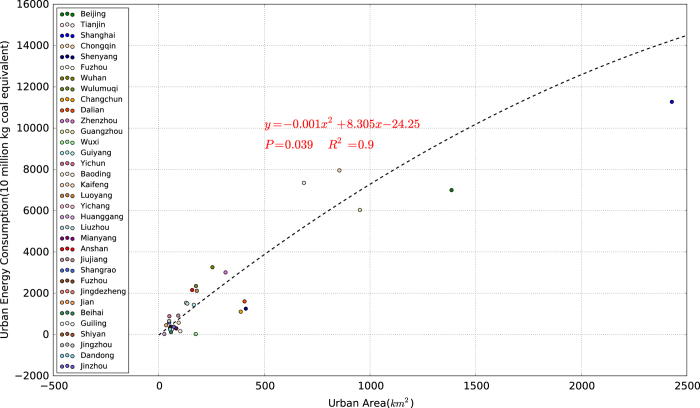
Relationship between urban areas and energy consumption For a total of 35 red points representing urban areas and their corresponding energy consumption (statistical data can be accessed in Supplementary File 1).

The algorithm for calculating the global anthropogenic heat product (AH-DMSP) developed in this study is presented in a flowchart (Fig. 1). First, we extracted the urban areas from calibrated DMSP-OLS and MODIS NDVI from 1992 to 2010 (details in step 1 and 3). Second, for each country, we separated the primary energy consumption (coal, petroleum, natural gas, and renewable energy) into urban and non-urban consumption using the urban consumption ratio from the International Energy Agency (IEA) (details in step 2). Third, a strong exponential relationship between urban area and energy consumption (*R*^2^=0.9, *P*=0.039) was found from the available statistical data (Fig. 2). Based on this relationship, the energy consumption in urban areas obtained from Step 2 was inserted in the urban map obtained from Step 3. Most large factories or refineries are located in urban areas. Therefore, the energy consumed in an urban area not only includes the energy consumption for domestic use but also industrial usage. Human metabolism occupied a small share of the total heat released by human activities, and we therefore excluded it. We note that statistical errors in urban energy consumption are a function of urban area and energy consumption. In order to alleviate their effects, we can allocate the total urban energy into patches based on percent-normalized urban energy from Fig. 2. Finally, we downscaled the total urban energy consumption to the pixel-level using the corresponding spatial proxy obtained in Step 1. The rural energy consumption at the country level was allocated directly into each grid. Finally, the energy consumption value was divided by elapsed time, and the resulting AH-DMSP of each grid cell was converted to anthropogenic heat flux (AHF, W.m^−2^).

The calculation is simplified by the following balance equation:

$$A_{n}=\sum_{i=1}^{4}k_{i}.E_{ni}$$

where *A*_n_ is the total anthropogenic heat of country n (1, 224); *k_i_* is the conversion rate of type *i* energy to heat, in which type *i* refers to the four sub-types of energy consumption: coal, petroleum, natural gas, or renewable energy, and E_*ni*_ is the energy consumption of type *i* for country *n*. The values for *k* were obtained from the EIA (http://www.eia.gov/beta/international/data).

### Step 1: Calibrating DMSP/OLS: Saturation and temporal fluctuation

Noise caused by less important persistent lighting sites such as gas flares, and ephemeral events such as fires in DMSP/OLS, occur mainly in the desert regions far from human habitants, but may affect anthropogenic heat precision. Here we masked out areas with a gridded population density less than 1 per 2.5 arc-minutes with the assumption that they would have no release of anthropogenic heat.

There are two drawbacks in the DMSP/OLS data: inter-annual variation and digital saturation in downtown areas^31–34^. Due to the auto-calibration system on the remote sensors, DMSP/OLS composites from different years cannot be inter-compared^31^. To address this problem, we employed the method developed by Elvidge^32^, using a second-order polynomial function to calibrate the annual fluctuation of DMSP/OLS data. Saturation values in DMSP/OLS data largely restrict the capacity of characterizing variations of inner urban region. We used a new spectral index to correct the saturated pixels, the Vegetation Adjusted Normalized Urban Index(VANUI) developed by Zhang^35^, based on the assumption that vegetation abundance is closely and inversely correlated with the intensity of energy consumption in urban areas.

### Step 2: Estimating ratios of urban energy consumption to total national consumption

The International Energy Agency (IEA) only calculates the ratios of urban energy consumption to total national consumption for four regions: United States, European Union, Austral-Asia, and China (Data Citation 4). In addition to these four regions, we introduce a simple method to estimate the urban energy consumption ratios for other countries or regions. First, 224 countries were grouped into two classes: high-income and low-income countries (including middle-income countries) based on gross national income (GNI) per capita. Second, the average urban energy consumption ratios from the United States, European Union, and Australia were applied to the high-income countries, while the ratio from China was applied to the other countries. The urban and rural energy consumption for each country could then be separated.

### Step 3: Extracting urban boundaries by support vector machines (SVM)

The existing global urban datasets have been applied for many studies of climate change and ecology (e.g., the Global Rural Urban Mapping Project or MODIS 500 m urban extent maps)^36^, but their temporal resolutions do not meet our requirements. Many previous studies indicate that the SVM algorithm^31,34,37^, which is widely used for classification and regression analysis, performs well at monitoring urban patches. It has the advantage of improving the accuracy of classification by encompassing substantial information, such as economic activities and urban vegetables^38^. Moreover, this method is not sensitive to the initial training samples, and it has the flexibility of multi-temporal and multi-spatial analysis^39^.

Here, we use the annual mean NDVI and calibrated DMSP/OLS as input information to the SVM-based algorithm, and extracted the urban boundaries for every year. We set the threshold (excluding water pixels inside the urban boundary) at DMSP/OLS >30 and NDVI <0.3 for developing countries^39^, and at DMSP/OLS >53 and NDVI <0.3 for developed countries. The Gaussian radial basis function (RBF, exp(−*γ*|*μ*−*ν*|^2)) was used as the kernel function, with the coefficient of cost (−c=2) set to 2 and gamma was 0.02 (−g=0.02). Noise in DMSP/OLS can be caused by a variety of reasons, such as variability in the atmosphere interference, and may cause errors in the boundary extraction output. Due to the stability of urban patches, we assumed that any urban patch detected in an earlier year by DMSP/OLS should be maintained in the results of later urban boundaries. In the end, we generated urban boundary data for each year during the period 2001–2012.

### Step 4: Estimating energy consumption for all urban patches

When a city starts expansion, it normally means its urban population grows and energy consumption grows accordingly. However, when a city reaches a mature stage of economic development and infrastructure construction, energy consumption will grow relatively slower than its expansion due to increase of energy efficiency. City energy data are difficulty to find and the difficult of acquiring urban energy data lies in consideration of city boundary issues (Data Citation 4). Therefore, we try to interpret energy consumption with city size in this study. The correlation parameters are developed with statistical data in China, which is provided in Supplementary File 1.

There was a strong exponential relationship between the urban area and energy consumption (y=−0.001x^2^+8.30x−24.25, *P*=0.039, where y is the urban energy consumption (10 million kg) and x is the urban magnitude (km^2^) (Fig. 2). This relationship was then applied to extract energy consumption for each urban patch from the total national urban energy consumption calculated in 2.2.2 and 2.2.3.

It is common practice to validate the accuracy of global-scale AHF estimation with anthropogenic heat data using a detailed statistical model, which consists of heat released from three components: the building sector, transportation, and human metabolism. The building sector is always divided into heat released from electricity consumption and from heating fuels, such as natural gas and fuel oil^40^. The detailed model focuses on the end-user of energy, while the inventory method focuses on primary energy. These methods can improve model accuracy by incorporating better information about individual cities^20^. For this study, the annual mean AHF estimated using our method was validated by AHF data estimated using the detailed model at multiple scales, from the city to province level. These data were estimated using the detailed statistical model and were compared with zone averaged AHF from the AH-DMSP for specific cities or provinces. Meanwhile, inter-validations of spatial patterns and single points for specific cities were also performed with previously created global gridded datasets. These annual mean global datasets were calculated by the inventory approach, and single points for specific AHF data were zone averaged.

### Code availability

The algorithm is coded in Matlab and C. AHF.m is the main procedure to calculate AH-DMSP. The global urban dataset is produced by Global_urban.m and its core parts are opened source code which are provided by Chang and Lin (http://www.csie.ntu.edu.tw/~cjlin/libsvm). Areas of global urban patches are calculated by Recursion.c and only binary files can be inputted and outputted in the procedure (write_binary.m and write_tif.m are designed for converting file format between binary and tiff). ArcGIS and Matplot or Python are exploited to draw figures. This code is available alongside the dataset at figshare (Data Citation 4).

## Data Records

### AH-DMSP dataset

AH-DMSP is a global gridded dataset of annual mean anthropogenic heat and its spatial resolution for the entire planet is 1×1 km^2^. Tagged image file format (TIFF) and network common data form (NetCDF) of AH-DSMP are provided in the repository (Data Citation 4). For the TIFF formats of AH-DMSP, its tfw format file is about spatial information of longitude and latitude for corresponding file. These data can be processed by GIS software, Matlab, Ncl or R etc. The uploaded data includes AH-DMSP at 1992,2001 and 2010 and is stored in uint16, which can be converted to annual mean anthropogenic flux (W.m^−2^) by a factor 0.1 (Data Citation 4).

## Technical Validation

### Spatial and temporal patterns of AH-DMSP

**Figure 3:**
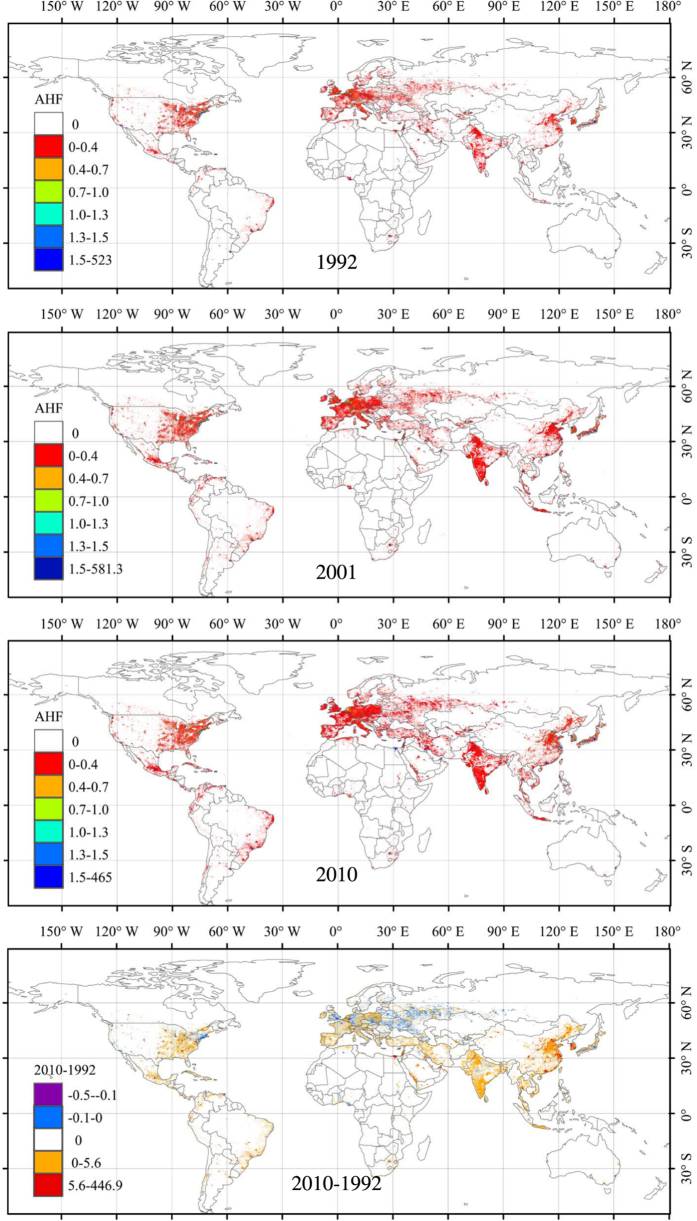
Temporal and spatial patterns of the annual mean AHF from 1992 to 2010. The bottom right figure,2010–1992, represents the differences in AHF between 1992 and 2010.

As shown in Fig. 3, spatial distributions of annual mean AHF are mainly in the developed regions, i.e., the eastern part of North America, Western Europe, and the eastern and southern parts of Asia. Obviously, the northern hemisphere contributes significantly more annual mean AHF than the southern hemisphere, which is consistent with the distribution of intensity and magnitude of human activities. All of the highest annual values appear in urban regions. Pixels with annual mean AHF greater than 1.59 W.m^−2^ occur in densely populated regions, and their numbers increase gradually with increasing global urbanization. The differences in spatial distribution of annual mean AHF between 2010 and 1992 are significant for the urban regions in rapidly developing countries, as well as in regions where there has been ongoing economic depression (for example, in Russia, Poland, and Ukraine).

**Figure 4:**
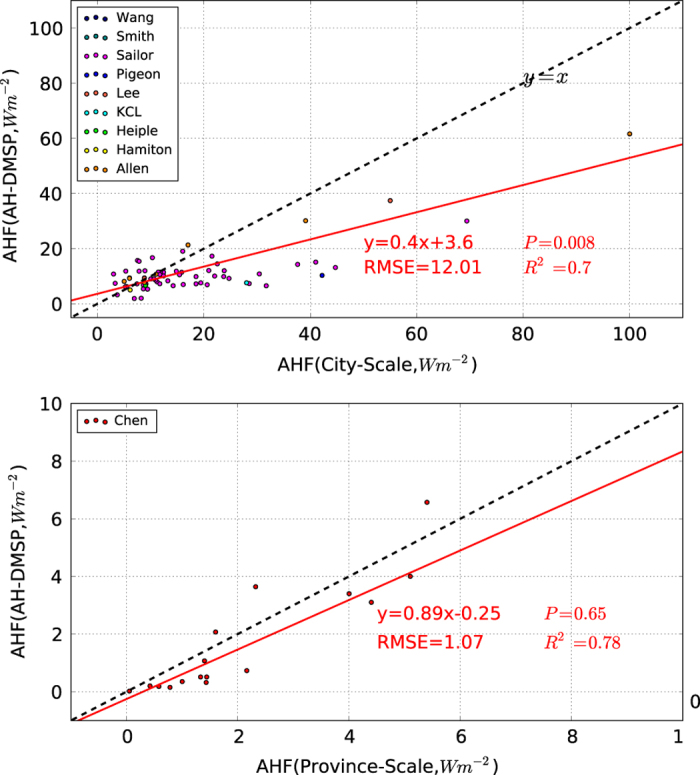
Validations of annual mean AH-DMSP with annual mean AHF-stat data at the urban and provincial scales. These data were obtained from^20,23,43–45^.

Figure 4 shows a comparison between the annual mean AHF from AH-DMSP and AHF data estimated with a detailed statistical model (AHF-stat) from the city to province scales. The statistical tests at the city and province scales between AH-DMSP and AHF-stat were *P*=0.008 and *P*=0.65, respectively. At the city level, AH-DMSP has good estimates of anthropogenic heat for cities whose AHF was less than 30 W.m^−2^. However, with urban developments, the differences between AH-DMSP and AHF-stat became increasingly significant. Root mean square error (RMSE: W.m^−2^) between the AH-DMSP and AHF-stat both the city and province levels were 12.01 and 1.07, respectively. At the province scale, due to limited data from AHF-stat, the statistical test was insignificant.

**Figure 5:**
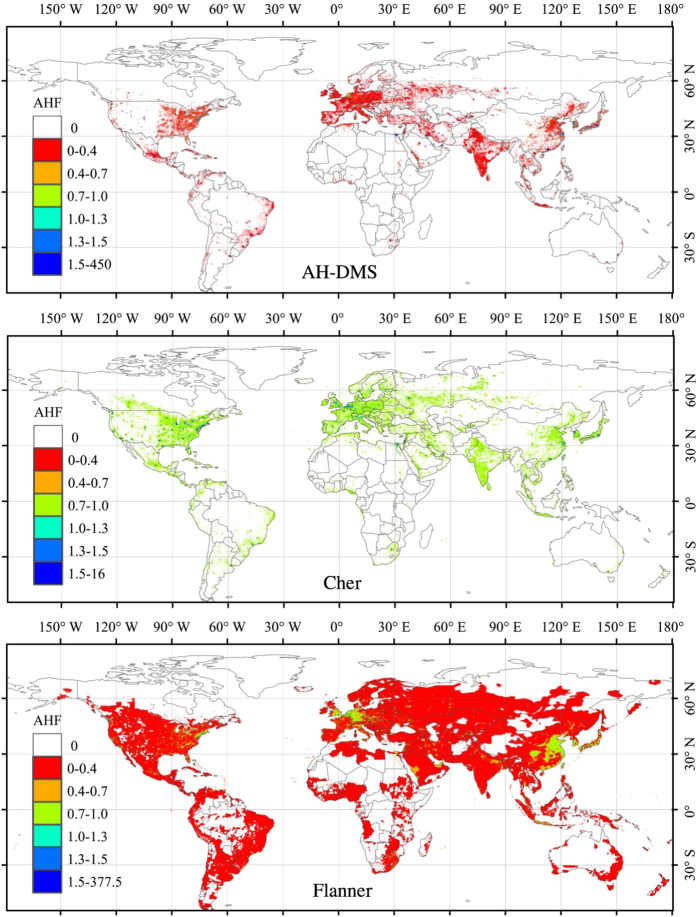
Annual mean AHF from three global datasets in 2005 from 1) AH-DMSP generated for this study, 2) data from Chen, and 3) data from Flanner; the scales for the three maps are uniform, to allow for direct comparison.

To assess the performance of AH-DMSP at the global scale, we made a comparison with two other global scale datasets from the studies by Chen and Flanner studies (Fig. 5)^20,21,41^. There are significant differences between the AH-DMSP, Chen, and Flanner data, both in the value ranges and the spatial patterns. Spatial patterns are quite similar between AH-DMSP and Chen’s data, since both studies use DMSP/OLS nighttime data, but the two have quite different value ranges. Most areas had values lower than 0.44 W.m^−2^ or higher than 1.31 W.m^−2^ in AH-DMSP, but the Chen data had values in the range from 1.02 to 1.31 W.m^−2^. This implies a significant influence from saturation, and intra- and inter-annual fluctuations of the DMSP/OLS data. Conversely, the Flanner dataset, which is currently used by the climate modelling community, had a larger area with AHF values, most of which are lower than 0.44 W.m^−2^.

**Figure 6:**
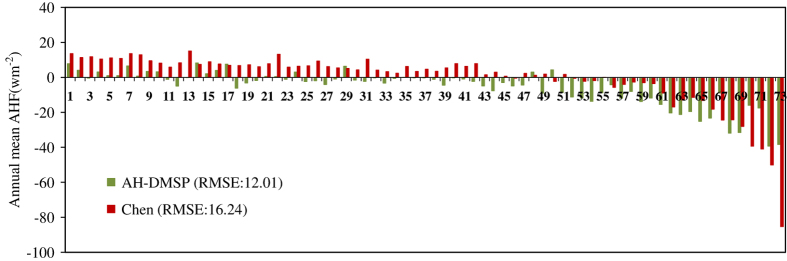
Biases of AH-DMSP and Chen data to AHF data Estimated with a detailed statistical model (X-axis represents the magnitude of cities, Y-axis represents the differences between a global dataset and AHF-stat; the city-level AHF data came from previous studies^11,16,20,24,40,42,44,45^); city names are replaced with numbers in Table 1 and detailed information about cites can be found in the Supplementary File 2.

We make comparisons of the annual means from previous datasets of AHF and AH-DMSP using the AHF-stat results as the real values. Since it is difficult to access annual mean AHF-stat data corresponding to the study period, only the Chen and AH-DMSP datasets were parallel compared in this section. From Fig. 6, with a growth in city magnitude, there is a trend in biases between the global datasets and AHF-stat data, which shift from positive to negative values and become more significant (city names in Table 1). For small cities, although the AHF values from global datasets is underestimated, its values are similar to the AHF-stat (real) values. For mega cities, values are significantly overestimated. From Fig. 6, AH-DMSP is closer to the real value than the Chen data.

## Usage Notes

Although anthropogenic heat is only about 0.3% of the total energy transported to the extra-tropics by atmospheric and oceanic circulations, it could disrupt normal atmospheric circulation patterns and warm surface temperatures at the local and global scales^10^. Therefore, incorporating anthropogenic heat into climate models could improve the performance of simulations of surface climate warming^6,10^ and are beneficial for studying the impacts of increased urban heat on the concentration of precursor species (e.g., NOx and CO) and resident health, such as morbidity and mortality risk, in cities^7,31^.

However, detailed evaluation of the impact on climate is deterred by the limited precision and spatial and temporal resolutions of anthropogenic heat data. The DMSP/OLS data has provided an opportunity to produce an improved precision and resolution AHF dataset. The applied methodology has been validated by a detailed anthropogenic heat model at multiple scales, but there are still some needed improvements. The underlying assumption that all energy is converted into anthropogenic heat is unreasonable, since some energy consumed will be stored by buildings and converted to other forms of energy. This assumption could therefore lead to overestimation of anthropogenic heat. Due to difficulties in observation; it is difficult to establish ratios for converting energy consumption to anthropogenic heat.

The observation approach includes in situ eddy covariance observations^13^ and the energy budget residual approach^14–16^. In situ eddy covariance observations of anthropogenic heat are seriously constrained by site-specific challenges, particularly in urban regions (e.g., permissions to install towers and equipment, access to measurement sites, and dense human activities)^13^. These challenges make it even more complex to find a site suitable for instrument observations and distinguish human activity signals from background noise in the highly heterogeneous urban environment. Previous studies have exploited the energy budget residual to calculate anthropogenic heat^19^; however, some uncertainties are introduced by latent heat and storage heat. A statistical detailed model, such as the town energy budget (TEB) or urban canopy model^17^, were developed by accounting for the building materials and heights, as well as the equipment used in buildings or transportation^18,19^. Conversely, for global scale anthropogenic heat estimations, available data about building, transportation, and urban energy consumption limits the scope of application. The inventory approach is more efficient for large-scale studies than the other two, which are limited by their demand categories and resolutions for modelling. The inventory approach is widely used to estimate global-scale anthropogenic heat^12,13^.

In order to keep temporal consistency with DMSP/OLS data, the annual mean NDVI for year 2001 was extended back as constant values to 1992, in order to complement the period 1992–2001. The simplified method produces less influence on the method performance, because the spatial resolution of MODIS instruments (1×1 km) has a limited ability for detecting variations in urban vegetation and cannot detect decade-scale variations^35,39^. Gridded population density data for the year 2000 were used to mask out noises in DMSP/OLS during the study periods. We assumed that areas with a gridded population density value lower than 1 have no release of anthropogenic heat and that the boundary of human habitations had no significant changes during the study period.

Validation revealed that the values of AH-DMSP were underestimated for certain mega-cities. The underestimation might relate to the selected ratio of urban energy consumption to the national, and an allocating function that distributes the total urban energy proportionally to each urban patch. Due to limitations in available statistics for urban sizes and energy use around the world, only four regions have ratios of urban energy consumption—United States, European Union, Austral-Asia, and China. The ratios for other countries were calculated based on gross national income (GNI) per capita, which could have negative influences on the accuracy of estimated anthropogenic heat for mega cities in these countries. Previous studies have argued that the SVM-based algorithm is not sensitive to initial training samples. Therefore, threshold values of NDVI and DMSP/OLS were used for extracting global urban patches^42^, and uniform training samples and thresholds of NDVI and DMSP/OLS were utilized around the world during the study period. This is another reason for underestimation in mega cities. However, separating total energy consumption into two types, urban and non-urban, then further downscaling urban energy to each city in each country is a step forward for calculating a robust global AHF product.

## Additional Information

**How to cite this article:** Yang, W. *et al.* A new global anthropogenic heat estimation based on high-resolution nighttime light data. *Sci. Data* 4:170116 doi: 10.1038/sdata.2017.116 (2017).

**Publisher’s note:** Springer Nature remains neutral with regard to jurisdictional claims in published maps and institutional affiliations.

## Supplementary Material



Supplementary File 1

Supplementary File 2

## Figures and Tables

**Table 1:** Relationship between city and NO. for cities labelled by digital number.

**NO.**	**City**	**NO.**	**City**	**NO.**	**City**	**NO.**	**City**	**NO.**	**City**	**NO.**	**City**
**1**	Oklahoma City	**13**	Birmingham	**25**	London	**37**	Fresno	**49**	Louisville	**61**	Baltimore
**2**	Jacksonville	**14**	El Paso	**26**	Indianapolis	**38**	San Antonio	**50**	Toulouse	**62**	Gyeonggi
**3**	Lexington-Fayette	**15**	Tulsa	**27**	Raleigh	**39**	Stockton	**51**	Milwaukee	**63**	Miami
**4**	Houston	**16**	Fort Worth	**28**	Riverside	**40**	Omaha	**52**	Pittsburgh	**64**	Philadelphia
**5**	Nashville-Davidson	**17**	Tucson	**29**	Phoenix	**41**	Columbus	**53**	Oakland	**65**	Boston
**6**	Colorado Springs	**18**	Tampa	**30**	San Diego	**42**	Portland	**54**	Cleveland	**66**	Chicago
**7**	Salt Lake City	**19**	Charlotte	**31**	Denver	**43**	Atlanta	**55**	Buffalo	**67**	San Francisco
**8**	Kansas City	**20**	New Orleans	**32**	Vancouver	**44**	San Jose	**56**	Los Angeles	**68**	Washington
**9**	Bakersfield	**21**	Memphis	**33**	Austin	**45**	Dallas	**57**	St Louis	**69**	San Francisco
**10**	Manchester	**22**	Wichita	**34**	Helsinki	**46**	Houston	**58**	Detroit	**70**	Incheon
**11**	Greater Manchester	**23**	London	**35**	Sacramento	**47**	Cincinnati	**59**	Seattle	**71**	Seoul
**12**	Corpus Christi	**24**	Albuquerque	**36**	London	**48**	Las Vegas	**60**	Minneapolis	**72**	New York
										**73**	Tokyo
